# Increased Host Investment in Extrafloral Nectar (EFN) Improves the Efficiency of a Mutualistic Defensive Service

**DOI:** 10.1371/journal.pone.0046598

**Published:** 2012-10-04

**Authors:** Marcia González-Teuber, Juan Carlos Silva Bueno, Martin Heil, Wilhelm Boland

**Affiliations:** 1 Department of Bioorganic Chemistry, Max Planck Institute for Chemical Ecology, Beutenberg Campus, Jena, Germany; 2 Departamento de Ingeniería Genética, CINVESTAV-Irapuato, Km. 9.6 Libramiento Norte, Guanajuato, México; Jyväskylä University, Finland

## Abstract

Extrafloral nectar (EFN) plays an important role as plant indirect defence through the attraction of defending ants. Like all rewards produced in the context of a mutualism, however, EFN is in danger of being exploited by non-ant consumers that do not defend the plant against herbivores. Here we asked whether plants, by investing more in EFN, can improve their indirect defence, or rather increase the risk of losing this investment to EFN thieves. We used the obligate plant-ant *Acacia-Pseudomyrmex* system and examined experimentally in the field during the dry and the rainy seasons how variations in EFN secretion are related to (i) ant activity, to (ii) the ant-mediated defence against herbivores and (iii) the exploitation of EFN by non-ant consumers. Extrafloral investment enhanced ant recruitment and was positively related to the ant mediated defence against herbivores. The ant-mediated protection from exploiters also increased in proportion to the nectar sugar concentration. Although the daily peak of EFN production coincided with the highest activity of EFN thieves, *Pseudomyrmex ferrugineus* ants protected this resource effectively from exploiters. Nevertheless, the defensive effects by ants differed among seasons. During the dry season, plants grew slower and secreted more EFN than in the rainy season, and thus, experienced a higher level of ant-mediated indirect defence. Our results show that an increased plant investment in an indirect defence trait can improve the resulting defensive service against both herbivores and exploiters. EFN secretion by obligate ant-plants represents a defensive trait for which the level of investment correlates positively with the beneficial effects obtained.

## Introduction

Plants have evolved multiple strategies to defend themselves against herbivores. They can either directly reduce herbivore attack through the production of chemical and physical defences [1], [2] or indirectly by producing rewards that attract the natural enemies of herbivores [3]. Most commonly reported in the latter context are herbivore-induced volatile organic compounds (VOCs) and extrafloral nectar (EFN) [4]. Even though the role of direct and indirect defences has been widely demonstrated, it remains an open question for most defensive traits how variations in the investment into a given trait translate into variations in its defensive effect [5]. A higher concentration of glucosinolates and a greater density of trichomes in genetic lines of *Arabidopsis thaliana* reduced the herbivory by two specialist flea beettles [6]. Similar quantitative effects have also been reported for the cyanogenic glyosides of lima bean (*Phaseolus lunatus*) [7]. Nevertheless, other studies have not found an enhanced resistance to specialist and generalist herbivores due to a higher concentration of secondary compounds [8]–[11]. Thus, it remains open for most defensive secondary compounds whether an increased herbivore pressure can exert a positive selective pressure on their quantitative levels.

This problem might be even more pertinent for indirect defences, which are achieved by interactions of plants with members of the third trophic level. These plant-carnivore interactions are mainly maintained by the provisioning of rewards to the defending carnivores [12]. However, such mutualistic interactions are also prone to exploitation [13]. Does, under these circumstances, a higher investment into the reward cause a better protection by ants? Or, in the context of plant-ant mutualisms: can ants defend their food rewards against exploiters and at the same time efficiently fulfil their mutualistic role in the indirect defence? In the present study we used an obligate plant-ant system to investigate whether an increased rate of EFN secretion improves the defensive effects of EFN or rather its exploitation by non-defending ‘EFN thieves’. EFN is rich in carbohydrates and amino acids that usually function in the attraction of ants and contains PR-proteins as protection from microbial infections [14], [15]. Ants that are attracted to EFN can effectively reduce the rates of herbivory of the host plant in nature [12], [16], [17]. In fact, EFN represents one of the few defensive traits for which positive effects on plant fitness have been unambiguously shown in natural ecosystems [5], [18]. Nevertheless, due to its high content in sugars, amino acids and even lipids [19]–[21] and because it is openly offered on the plant surface, EFN is also attractive to non-ant consumers such as bees, flies, mites, wasps and beetles [22]–[25]. The consumption of EFN by non-defending animal groups might represent for the plant an important loss of energy and other, potentially more limiting resources. Loss of EFN to thieves can even represent a reason for which some studies have failed to find an effective defence effect of ants attracted to EFN [22], [26]–[28]. Furthermore, exploitation of EFN by non-defending consumers would reduce the overall efficacy of this indirect defence and cause considerable indirect costs [29].

In the present study we used two *Acacia* species that are engaged in obligate defensive mutualisms with ants of the genus *Pseudomyrmex*. We used this system to investigate during both the rainy and the dry season, (1) whether a higher rate of EFN secretion is positively related to higher ant recruitment and a more effective defence by ants against herbivores, (2) a change in the relative investment in defence v/s growth, and (3) whether higher rates of EFN secretion affect the level of exploitation by EFN thieves. Taken together, the results allow us to elucidate whether increased investment into EFN leads to an enhanced efficiency of the resulting defensive effect.

## Materials and Methods

### Plant material and study sites

We investigated two myrmecophyte species in April–May 2010 (dry season) and in September–October 2010 (rain season): *Acacia cornigera* (L.) Willendow and *Acacia hindsii* Benth., which differ in their host quality with respect to the natural patterns of EFN production [30]. Myrmecophyte *Acacia* plants secrete EFN as a constitutive trait to nourish their ant colonies (*Pseudomyrmex ferrugineus* F. Smith and related *Pseudomyrmex* species), and the ants are nutritionally dependent on their host plant [31]. Ants live in domatia and collect EFN and food bodies. In return, the ants effectively defend their host plants against herbivores [4], [32]. Several species have been described as exploiters of these systems: they make use of the plant-derived resources without rendering a defensive service [31], 33–36.

All plants used were growing naturally in the coastal area of the state of Oaxaca (México), 15 km northwest of Puerto Escondido (Pacific coast; ∼15°55′N and 97°09′W, elevation 15 m). All plants were shrubs 1.5–2.5 m high and grew in the full sun. Species were determined following Janzen [37] and Seigler & Ebinger [38] and by comparison with specimens held at the Herbario MEXU at UNAM (Mexico City), ant species were determined following Ward [39]. The climate in the zone is characterized by one main rainy season from July to October, which follows a bimodal distribution peaking in July and September. Annual rainfall averages between 1000 and 1400 mm and the average temperature is 28°C [40], [41]. The months from November till June are characterized by a hot and dry climate, no rain and temperatures up to 40°C (personal observations).

### EFN quantification and ant activity

The collection and quantification of EFN was conducted as follows. Branches of *Acacia* host plants were deprived of ants and other insects the day before nectar collection, by cutting off the inhabited thorns, mechanically removing ants and then placing the branch in a mesh bag after isolating it from the rest of the plant by applying a ring of sticky resin (Tangletrap, The Tanglefoot Corp. Grand Rapids, Mich., USA). After one day, nectar production rates of the first five new leaves were quantified as amounts of soluble solids per 24 h and per gram leaf dry mass, by quantifying the nectar volume with micro capillaries (Hirschmann Laborgeräte GmbH & Co. KG, Eberstadt, Germany) and the nectar concentration with a refractometer (Atago Co. LTD.) as described previously [42]. The leaves that had produced the EFN were then collected and dried (50°C for 48 h). EFN was collected from 13–17 individuals from each species in each season.

The activity of the resident ant *P. ferrugineus* F. Smith was determined on the same branches from which EFN was collected, one day before EFN quantification. Three equally distant lines were drawn along the branch. Lines were drawn 24 h before the experiment to exclude any putative effects of odours released from the ink on ant behaviour. Ant activity was evaluated as the number of ants that crossed at least one of the lines during one minute. All experiments evaluating ant activity were carried out between 10:00 and 13.00 because this time of the day represents the phase with the highest activity of *P. ferrugineus*. Effects of season and species (independent variables) on EFN production and ant activity (dependent variables) were evaluated using a factorial design two-way ANOVA. A Tukey HSD test was used for *a posteriori* comparisons.

Individual plants were the units of replications and the sample size were the following: *A. cornigera* – dry season: *n* = 17, *A. cornigera* – rainy season: *n* = 12, *A. hindsii* – dry season: *n* = 16, *A. hindsii* – rainy season: *n* = 13. The relationship between EFN production and ant activity per individual was then evaluated for each *Acacia* species with a Pearson's correlation coefficient.

### Ant recruitment in response to different EFN concentrations

To determine if the recruitment of *P. ferrugineus* increases with increasing EFN concentration, three branches with similar conditions (position on the main shoot and number of leaves) per plant were selected in 10 individual plants of *A. cornigera* and *A. hindsii* in April 2010. We applied 10 µL of an artificial nectar mimic near the EFN nectaries on each of three youngest leaves of every branch, assigning the three branches of every plant randomly to each one of the following treatments: 1) water, used as a control, 2) a 5% sugar solution (fructose∶glucose, 1∶1), 3) a 20% sugar solution (fructose∶glucose, 1∶1). The 1∶1 ratio of fructose∶glucose was used because it mimics the sugars found in EFN of myrmecophyte *Acacia* plants [21]. The concentrations of the artificial nectars were adjusted using a portable refractometer. The concentration of 20% of soluble solids (w/v) was chosen since it represents the normal concentration of natural, undiluted EFN of *Acacia cornigera* during the dry season. The normal concentration of *Acacia hindsii* is lower than *A. cornigera* and ranges between 5–15%. Nevertheless the concentration of EFN of *Acacia* species significantly varies depending on the season (see below). After the treatment application (at time 0), ant recruitment to the respective part of the branch was quantified as the total number of ants present on the new three youngest leaves after 1, 5, 10 and 20 min. A mixed model analysis was separately applied for each plant species. Since the treatments were applied on three branches of the same individuals, the individual plant was considered as a random factor. Sugar solution was considered as fixed factor and time as fixed and as random factor. Since the ant recruitment did not show a linear response over time (see below), different time points were considered as separate factor levels. The model was fitted by REML (Restricted Maximum Likelihood Method). The individual plants were the units of replications with a sample size of 10 individuals for each plant species.

### Ant activity and ant aggression

To test if the intensity of ant aggression increases with increasing EFN concentration, two branches of a same individual plant (with similar conditions: position within the plant, number of leaves and thorns) were selected in 10 plants of *A. cornigera* and *A. hindsii* in September 2010. 10 µL drop of the two following treatments: 1) 5% sugar solution (fructose∶glucose, 1∶1), 2) 20% sugar solution (fructose∶glucose, 1∶1) was applied at the base of each of all leaves along the entire branch. Before ant aggression evaluation, we quantified ant activity (by counting the number of ants crossing lines marked along the branch) for each of the treatments as described above. Ant aggression was evaluated by fixing three larvae of termites (of the same species) with a needle on the central part of each branch twenty minutes after the application of the different sugar solutions. This time was chosen because 20 min was the time at which ant number recruited to the nectar droplets peaked (see below, ant recruitment results). Aggression against ‘enemies’ was determined as the number of ant attacks ( = number of ant bites) to the termites over the first three minutes after placing the termites. It has been shown that termites are able to elicit a response in ants [43] and that the presence of a foreign insect, together with the mechanical damage produced by the needle, represents a suitable enemy mimic. A mixed model analysis was used to determine the effects of the plant species and sugar solution (fixed factors) on the ant activity and on ant aggressiveness. Since the treatments were applied on two branches of the same individuals, the individual plant was considered as a random factor. The model was fitted by REML. The plant was the units of replications with a sample size of 10 individuals for each plant species.

### Natural herbivory and growth of *Acacia* plants

To determine natural leaf damage in both *Acacia* plants, ant exclusion experiments were carried out using 10 *A. cornigera* and 10 *A. hindsii* plants each during the dry season (March–May) and the rainy season (September–November). Ants of *P. ferrugineus* were excluded for one month from one branch of each of the individual plants (as described above). A second branch was selected as a control, which was the most similar one to the ant exclusion branch with respect to its position within the plant and the number and quality of leaves. Thorns were also removed from control branches in order to avoid potential damaging effects of cut thorns on plant leaves. After one month, three leaves were randomly selected from each of the two experimental branches (ants present and ants absent) per plant, in order to determine leaf damage levels. Leaf damage was determined counting the total number of leaflets and damaged leaflets to calculate the percentage of damage. Effects of the season, plant species and presence/absence of ants on the percentage of damaged leaflets (‘herbivory’, as dependent variable) were evaluated with a mixed model analysis. Season and presence/absence of ants were considered fixed effects whereas the individual plant was considered a random effect. The model was fitted by REML. Herbivory data were arcsine-transformed prior to analysis. Individual plants were the units of replications with a sample size of 10 plants for each plant species.

Using the same plants as those evaluated for EFN quantification and ant activity, we determined the growth rate of *Acacia* plants in both seasons (sample size: *A. cornigera* – dry season: *n* = 14, *A. cornigera* – rainy season: *n* = 12, *A. hindsii* – dry season: *n* = 15, *A. hindsii* – rainy season: *n* = 12). Plant growth rate was estimated by carefully attaching a marker at the top of a neighbouring branch to those selected for EFN quantification and measuring the biomass (g) of the total new leaves produced after one month. Effects of the season and plant species on the total biomass produced per month (g) (dependent variable) were evaluated using a two-way ANOVA (individual plants as units of replications) with a Tukey's HSD post hoc test. Data were log transformed to achieve homogeneity of variance.

### Diurnal patterns in EFN production and ant activity

Diurnal patterns in EFN production and ant activity were quantified for five plants each of *A. cornigera* and *A. hindsii* (April 2008). The main branch of every plant was deprived of resident ants the day before nectar collection as described above. EFN was collected from the three youngest fully developed leaves on the main branch, every 2 h from 8.00 until 22.00. Before the first nectar collection (at 6.00), nectaries were washed with distilled water to remove any accumulated nectar. EFN secretion was quantified as amounts of soluble solids per gram leaf dry mass as described above (sample size of 5 individuals for each plant species). At the same time and on the same individual plants from which EFN had been collected, activity of the resident ants was determined (as described above). The relationship between EFN production and ant activity across time was then evaluated for each *Acacia* species with a Spearman rank correlation test, using the means for EFN production and ant activity calculated for every time of the day from the values of all five individuals per species.

### Ant activity and protection from EFN thieves

Protection of EFN from thieves by resident *P. ferrugineus* ants was evaluated through ant exclusion experiments, which were carried out as described above. Two treatments, (i) three branches without ants (ant-free) and (ii) three branches with ants (ants present), were applied to each plant. The activity of EFN thieves was determined as the number of insects landing on leaves of these experimental branches during 60 sec and was censused every 2 hours from 08.00 to 22.00. The only group of animals showing up as EFN thieves were bees, which were identified by Dr. Roubik, Smithsonian Tropical Research Institute, Balboa, Ancon, Republic of Panama. Differences in the activity of EFN thieves between ant-exclusion branches and control branches over time were evaluated with a mixed model analysis (individual plants were units of replication, *n* = 5 for each plant species). Since EFN thieves were only active from 08.00 to 10.00 AM (see below), only these two time points were included in the analysis. Time and presence/absence of ants were considered fixed effects whereas the individual plant was considered a random effect. The model was fitted by REML.

A second experiment was carried out to evaluate whether the ant aggression towards thieves increased in proportion to the sugar concentration in the EFN (September 2010). Two branches with similar characteristics (position at the plant, number of leaves and thorns) were selected from 10 plants of *A. cornigera*. 10 µL drops of a 5% artificial sugar solution were applied at the base of each leaf along one branch. On the second branch 10 µL drops of a 20% artificial sugar solution (fructose∶glucose, 1∶1) were also applied at the base of each leaf along the branch. The intensity of aggression towards thieves by mutualistic ants was determined for each branch ten minutes later as the number of ant attacks ( = number of ant bites) to the EFN thieves that landed on the extrafloral nectaries during three minutes. Protection by ants was evaluated after 10 min since we had previously shown that the recruitment of *P. ferrugineus* peaks at 10 min after the application of artificial nectar (see below). The time (seconds) of duration of visits by individual EFN thieves was also evaluated on both branches of all of the 10 selected plants that had been treated with the different artificial sugar solutions. Significant differences in ant attacks to EFN thieves and visit duration by EFN thieves between both branches (5% sugar solution v/s 20% sugar solution) from the same individual plant were evaluated with a Wilcoxon test for matched pairs (plants as units of replications, *n* = 10 plants). All the analyses were done with SPSS Statistics 17.0.

### Ethics statement

Since all experiments were not conducted on private grounds, specific permissions were not required for the activities. *Acacia* plants and ants did not involve endangered or protected species.

## Results

### EFN production and ant activity

**Figure 1:**
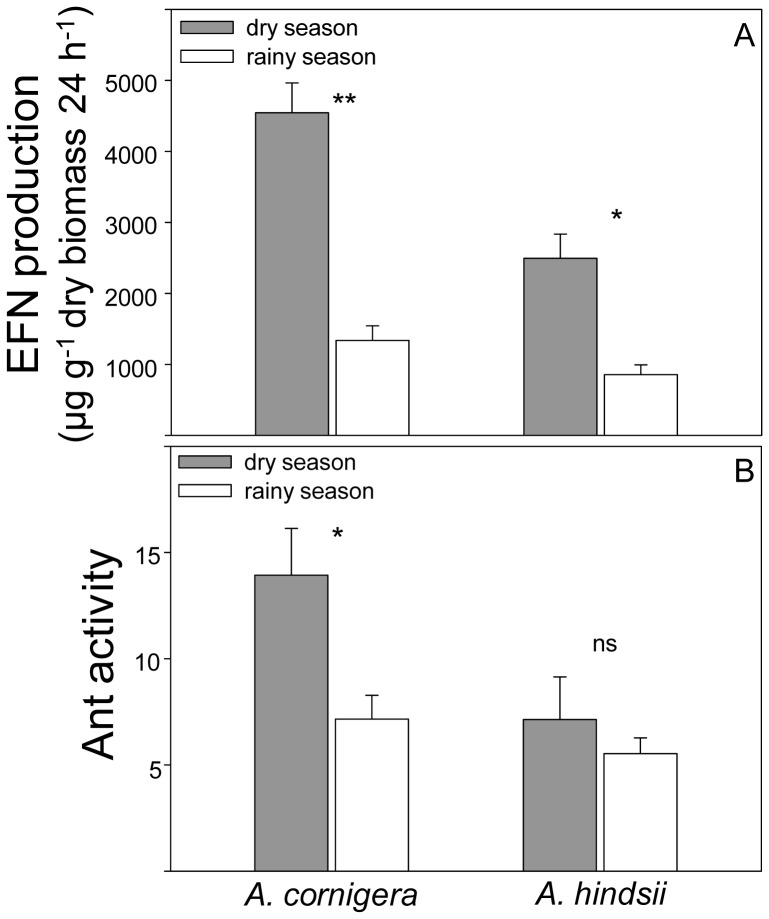
Changes in EFN production and ant activity during the dry and the rainy season. (A) EFN secretion pattern (µg g^−1^ dry biomass 24 h^−1^) and (B) the number of ants that crossed three lines along the plant branch during three minutes in *A. cornigera* and *A. hindsii* during the dry (gray bars) and the rainy season (open bars). Data represent mean and standard errors. Asterisks indicate significant differences (*** *P*<0.001, ** *P*<0.01, * *P*<0.05, ns = not significant, Two-way ANOVA).

EFN production was significantly affected by the season (*F*_1,54_ = 37.1, *P* = 0.000, Two-way ANOVA) and by the plant species (*F*_1,54_ = 7.91, *P* = 0.006, Two-way ANOVA). It was higher in the dry season than in the rainy season for both plant species and it was significantly higher for *A. cornigera* than for *A. hindsii* (Fig. 1A). No significant ‘season×plant species’ interaction was found for EFN production (*F*_1,54_ = 2.71, *P* = 0.100, Two-way ANOVA). A similar pattern was observed for ant activity, which was significantly affected by the season (*F*_1,54_ = 7.30, *P* = 0.009, Two-way ANOVA) and by the plant species (*F*_1,54_ = 7.38, *P* = 0.008, Two-way ANOVA) (Fig. 1B). No significant ‘season×species interaction’ was found for ant activity (*F*_1,54_ = 2.76, *P* = 0.100, Two-way ANOVA). EFN production and ant activity were significantly correlated for *A. cornigera* in the dry season and marginally significant in the rainy season (*A. cornigera* – dry season: *r* = 0.51, *P* = 0.023, *n* = 17; *A. cornigera* – rainy season: *r* = 0.53, *P* = 0.073, *n* = 12; Pearson's correlation coefficient). For *A. hindsii*, in both seasons the correlation was not significant (*A. hindsii* – dry season: *r* = −0.19, *P* = 0.431, *n* = 16; *A. hindsii* – rainy season: *r* = 0.35, *P* = 0.234; *n* = 13; Pearson's correlation coefficient).

### Ant recruitment in response to different EFN concentrations

**Figure 2:**
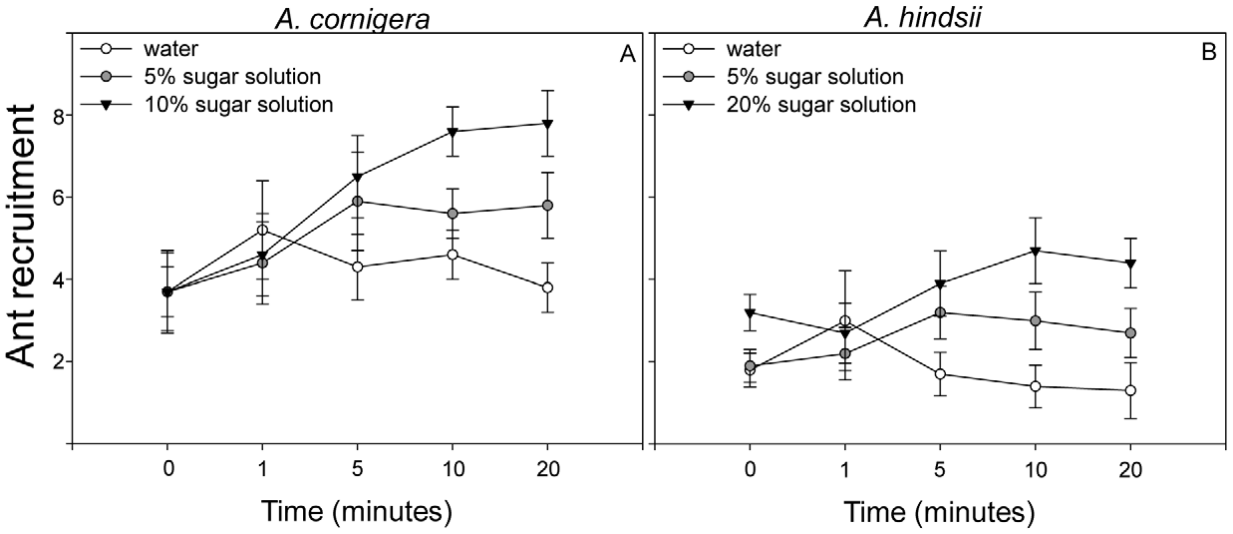
Effect of nectar concentration on time of course of ant recruitment on two *Acacia* species. Number of ants present on branches treated with different concentrations of artificial nectar solutions, water (black circle), 5% sugar solution (white circle) and 20% sugar solution (black triangle) for (A) *A. cornigera* and (B) *A. hindsii* (Mixed model analysis).

Ant recruitment on the host *A. cornigera* was significantly affected by the concentration of the sugar solution (*F*_2,126_ = 6.96, *P* = 0.001, Mixed model analysis) and by the time (*F*_4,126_ = 5.49, *P* = 0.000, Mixed model analysis). We also observed a significant ‘nectar concentration×time’ interaction (*F*_8,126_ = 2.02, *P* = 0.048, Mixed model analysis), which indicates that the ant response to the different sugar solutions varied significantly over the observed time span (Fig. 2A). In fact, a higher number of ants were recruited to those leaves that had been treated with a 20% sugar solution than to those treated with a 5% sugar solution or with water, but significantly so only 10 and 20 minutes after the onset of the experiment. For the plant species *A. hindsii* there was a significant effect of the nectar concentration (*F*_2,126_ = 19.70, *P* = 0.000, Mixed model analysis), but not of time (*F*_4,126_ = 0.890 *P* = 0.472 Mixed model analysis). A significant ‘nectar concentration×time’ interaction was also found for *A. hindsii* (*F*_8,126_ = 2.65, *P* = 0.010, Mixed model analysis), i.e., such as on *A. cornigera*, the differences in ant recruitment among the three sugar solutions were only significant after 10 and 20 min (Fig. 2B).

### Ant activity and ant aggression

**Figure 3:**
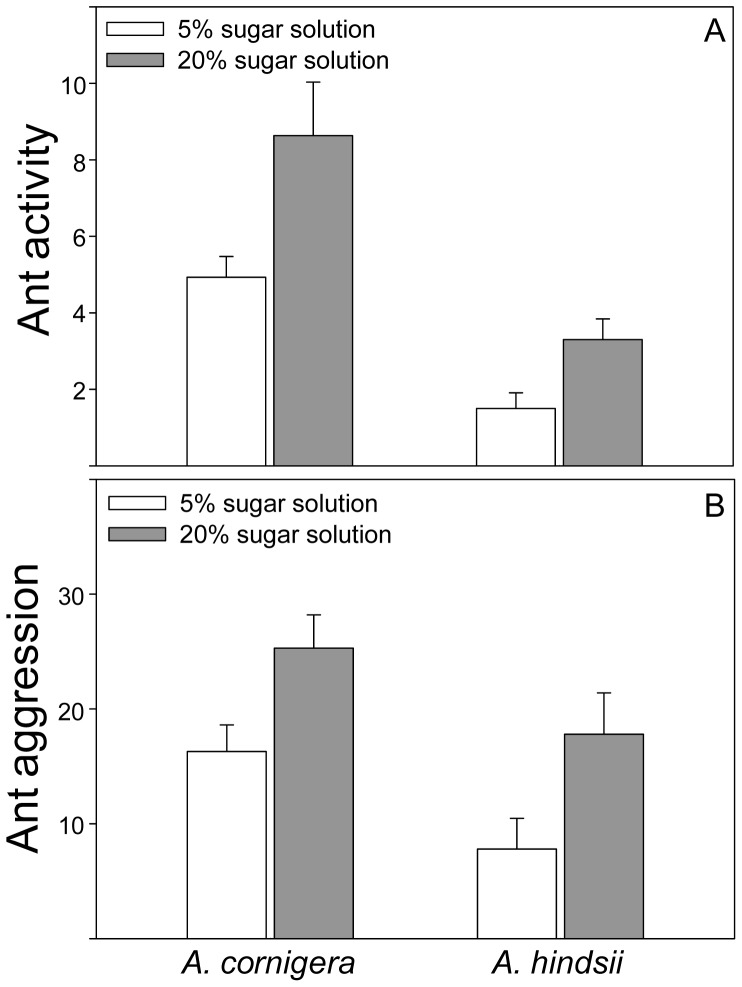
Effect of different nectar concentrations on ant activity and ant aggression on two *Acacia* species. (A) Ant activity (number of ants) and (B) ant aggression against termites (number of ant bites to termites placed on the host plant over three minutes) on branches of *A. cornigera* and *A. hindsii* treated with two different sugar solutions, 5% (white bars) and 20% (gray bars) (Mixed model analysis).

Ant activity was significantly affected by the plant species (*F*_1,18_ = 16.74, *P* = 0.001, Mixed model analysis) and by the concentration of the sugar solution (*F*_1,18_ = 28.17, *P* = 0.000, Mixed model analysis). A higher ant activity was observed in *A. cornigera* than in *A. hindsii* and on those branches that had been treated with the 20% sugar solution than on those treated with 5% sugar solution (Fig. 3A). No significant ‘plant species×sugar concentration’ interaction was found (*F*_1,18_ = 1.76, *P* = 0.201, Mixed model analysis).

The higher ant activity led to greater ant aggression. Ant aggression was also significantly affected by the plant species (*F*_1,18_ = 5.18, *P* = 0.035, Mixed model analysis) and by the concentration of the sugar solution (*F*_1,18_ = 6.08, *P* = 0.024, Mixed model analysis). It was significantly higher on *A. cornigera* than on *A. hindsii* and on branches treated with 20% sugar solution than with 5%. No significant ‘plant species×sugar concentration’ interaction was found for this trait (*F*_1,18_ = 0.24, *P* = 0.628, Mixed model analysis) (Fig. 3B).

### Natural herbivory and growth of *Acacia* plants

**Figure 4:**
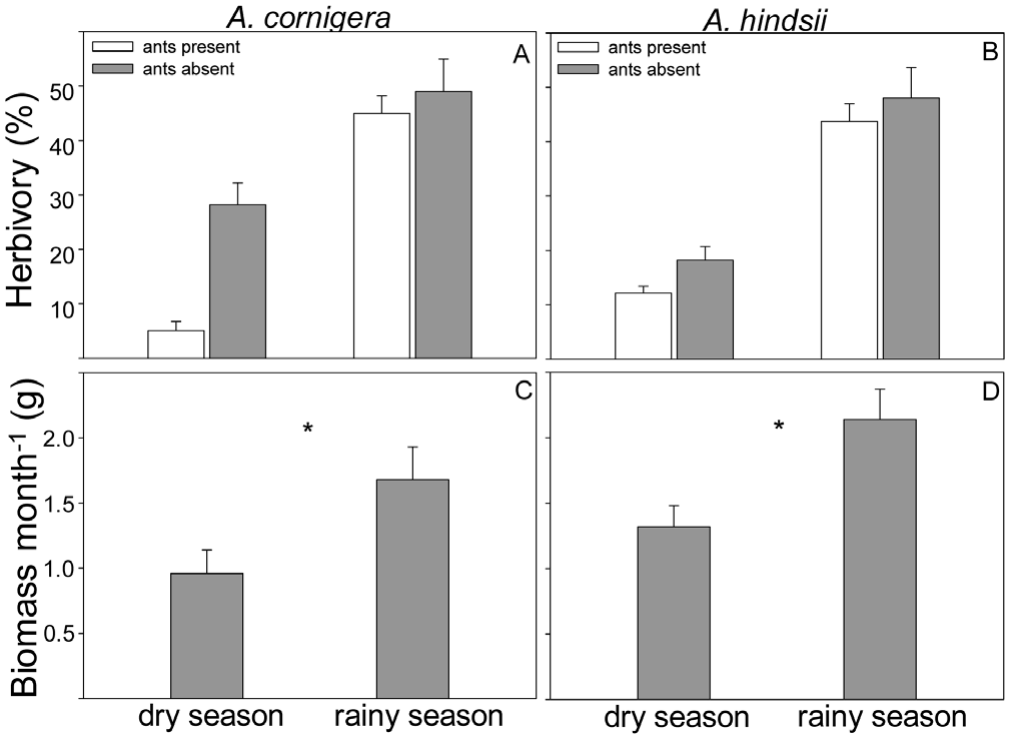
Herbivory levels and relative growth in two *Acacia* species during the dry and the rainy season. Percentage of damaged leaflets in branches of (A) *A. cornigera* and (B) *A. hindsii* in presence (gray bars) and absence (white bars) of ants of *P. ferrugineus* (Mixed model analysis). Biomass (g) produced after one month for plants of (C) *A. cornigera* and (D) *A. hindsii*. Asterisks indicate significant differences (*** *P*<0.001, ** *P*<0.01, * *P*<0.05, ns = not significant, Two-way ANOVA).

Herbivory was not affected by the plant species (*F*_1,36_ = 2.80, *P* = 0.103; Mixed model analysis), but it was significantly affected by the season (*F*_1,36_ = 191.0, *P* = 0.000; Mixed model analysis) and by the absence of ants (*F*_1,36_ = 91.4, *P* = 0.000; Mixed model analysis). For both plant species the herbivory was higher in the rainy season than in the dry season, but only in the dry season an ant-mediated defence against herbivores could be detected (‘season×absence of ants’ interaction: *F*_1,36_ = 52.38, *P* = 0.000; Mixed model analysis) (Figs. 4A, B). Furthermore, a significant ‘plant species×absence of ants’ interaction was also found (*F*_1,36_ = 9.75, *P* = 0.004; Mixed model analysis), suggesting that the absence of ants differentially affects the herbivory depending on the plant species. *A. cornigera* suffered higher herbivore attack than *A. hindsii* when the ants were removed from the plants. Thus, symbiotic ants defended better *A. cornigera* than *A. hindsii*.

Relative growth rate was significantly affected by the season (*F*_1,49_ = 8.80, *P* = 0.004, Two-way ANOVA) and by the plant species (*F*_1,49_ = 4.67, *P* = 0.035, Two-way ANOVA). Although the growth rate for both species was higher in the rainy than in the dry season, *A. hindsii* presented a higher growth rate than *A. cornigera* (Fig. 4C, D). No significant ‘season×plant species’ interaction was found (*F*_1,49_ = 0.39, *P* = 0.534, Two-way ANOVA). The difference in growth rate between season was caused by an increase in weight of leaves in the rainy season (*A. hindsii*: dry season = mean ± SE of five leaves for each individual: 0.28 g±0.01; rainy season = mean ± SE of five leaves for each individual: 0.51 g±0.03; *A. cornigera*: dry season = mean ± SE of five leaves for each individual: 0.18 g±0.008; rainy season = mean ± SE of five leaves for each individual: 0.34 g±0.01) (season: *F*_1,46_ = 28.1, *P* = 0.000; plant species: *F*_1,46_ = 59.0, *P* = 0.000; ‘season×plant species’ interaction: *F*_1,46_ = 2.39, *P* = 0.128; Two-way ANOVA). There were not differences in the number of leaves produced in both plant species between the dry and rainy season (season: *F*_1,46_ = 0.14, *P* = 0.707; plant species: *F*_1,46_ = 0.37, *P* = 0.541; ‘season×plant species’ interaction: *F*_1,46_ = 0.08, *P* = 0.766; Two-way ANOVA).

### Daily time course of EFN production and ant activity

**Figure 5:**
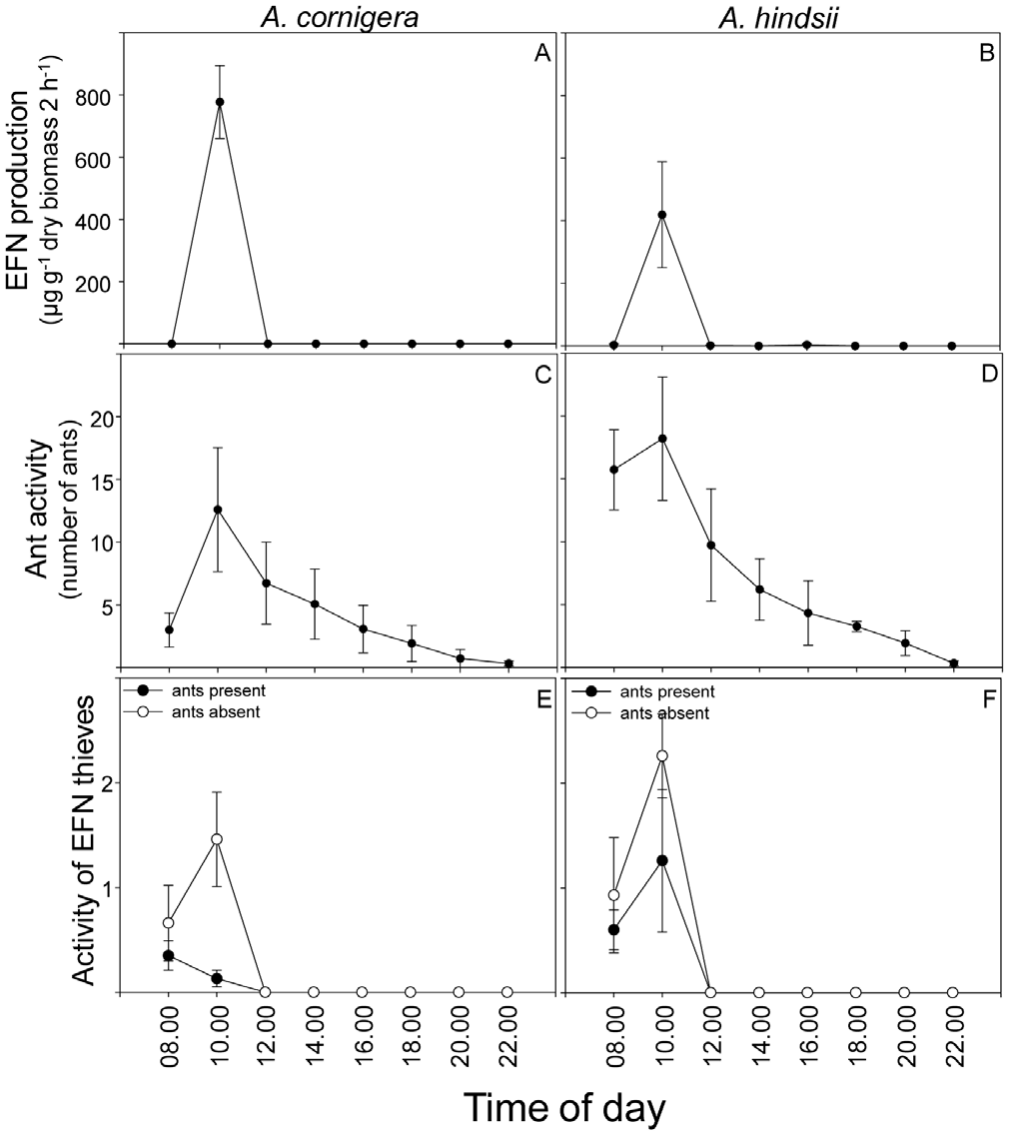
Daily time course of EFN production, ant activity and activity of EFN thieves. (A, B) Variations in EFN production (µg g^−1^ dry biomass 24 h^−1^) and (C, D) ant activity (number of ants) across the day for plants of *A. cornigera* and *A. hindsii*. Activity of EFN thieves was quantified in presence (black circle) and absence (white circle) of ants of *P. ferrugineus* on (E) *A. cornigera* and (F) *A. hindsii* (Mixed model analysis).

For both *Acacia* species, a significant and positive correlation was observed between the amounts of EFN produced and the ant activity on the respective plants (*A. cornigera*: *r* = 0.58, *P*<0.001: *A. hindsii*: *r* = 0.38, *P* = 0.030, Spearman rank correlation test). Moreover, the maximum activity of *P. ferrugineus* on the *Acacia* hosts coincided with the time of day during which peak EFN secretion could be observed (Fig. 5). For both *A. cornigera* and *A. hindsii*, the highest values of EFN production and ant activity were observed at 10.00 AM.

### Ant activity and EFN thieves

During our experiments, only one group of insect species was regularly observed as a nectar thieve on the two investigated *Acacia* species: bees of the species *Frieseomelitta nigra* (Apidae) (Cresson, 1878). EFN thieves only visited plants between 08.00 AM and noon (Fig. 5), which coincides mostly with the time of day for peak nectar production (Fig. 5A, B) and the highest activity of *P. ferrugineus* (Fig. 5C, D). *P. ferrugineus* efficiently protected the EFN from visits by *F. nigra* bees; however, the effect of ants was statistically significant only for *A. cornigera* (time: *F*_1,12_ = 1.15, *P* = 0.304; ant presence: *F*_1,12_ = 6.28, *P* = 0.028; ‘time×ant presence’ interaction: *F*_1,12_ = 2.05, *P* = 0.177; Mixed model analysis) (Fig. 5E). *F. nigra* visits to *A. hindsii* were not significantly different between ant-excluded and control branches (time: *F*_1,12_ = 1.69, *P* = 0.217; ant presence: *F*_1,12_ = 4.11, *P* = 0.065; ‘time×ant presence’ interaction: *F*_1,12_ = 0.75, *P* = 0.402; Mixed model analysis), although they showed a strong tendency to lower values on the control branches (Fig. 5F).

### Ant aggression towards EFN thieves

**Figure 6:**
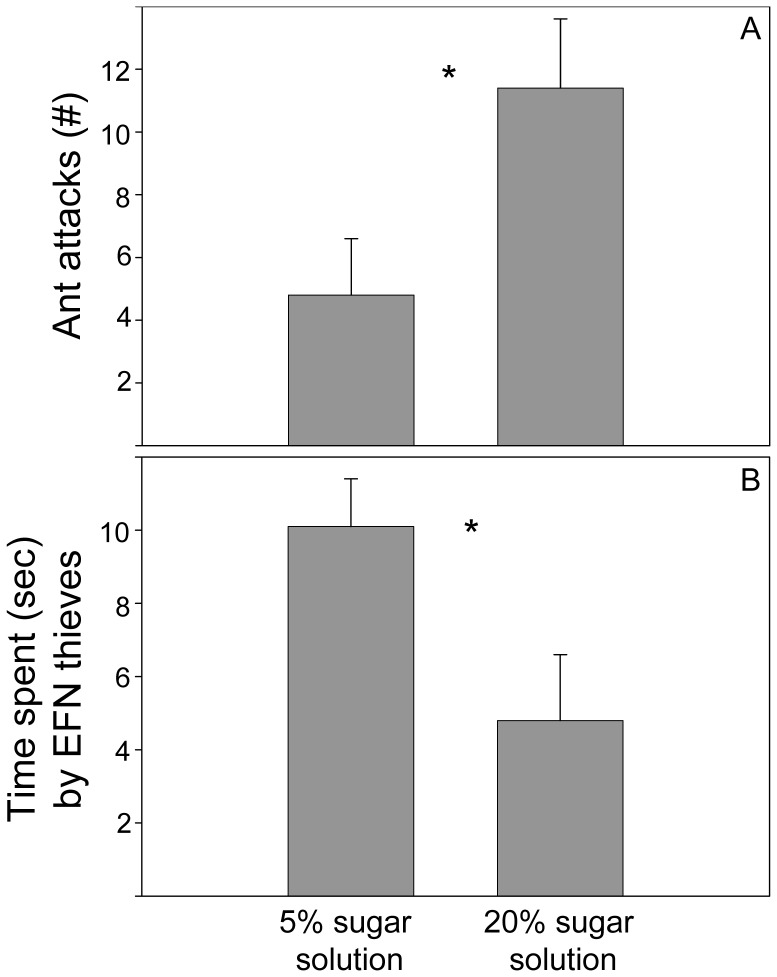
Aggression towards EFN thieves by ants of *P. ferrugineus*. (A) Number of ant attacks to EFN thieves and (B) time spent per visit (sec) by EFN thieves on branches treated with 5% and 20% of sugar solutions. Asterisks indicate significant differences in the number of bee visits between branches with and without ants at a certain time (*** *P*<0.001, ** *P*<0.01, * *P*<0.05, ns = not significant, Wilcoxon pair test).

The degree of ant aggression towards thieves depended on the nectar concentration. The number of ant attacks to EFN thieves was significantly higher on those plant branches that were treated with a 20% sugar solution than on those treated with the 5% sugar solution (5% = 4.8±1.8 number of ant attacks, 20% = 11.4±2.3 number of ant attacks, *Z* = 2.01, *P* = 0.04, Wilcoxon pair test) (Fig. 6A). The time spent per visit by EFN thieves on *Acacia cornigera* nectaries decreased significantly when the nectar concentration increased (5% = 10.1±1.3 seconds, 20% = 4.8±1.8 seconds, *Z* = 2.19, *P* = 0.02, Wilcoxon pair test) (Fig. 6B).

## Discussion

The primary aim of this study was to investigate whether an increase in the investment into an indirect defensive trait (extrafloral nectar, EFN) leads to an increased efficiency of the ant-mediated indirect defence against herbivores or rather to a higher risk of losing this investment to EFN thieves. Some studies on this topic have already assessed this relationship before, but only in terms of defence against herbivores [41], [44], [45]. Here we provide information about the effects of EFN investment on the risk of exploitation by nectar thieves and their effects on defence behaviour of symbiotic ants.

Our study revealed a very clear picture: a higher investment in EFN was related to a higher level of ant recruitment to the sites where the EFN was presented. Furthermore, ant activity of *P. ferrugineus* was related with better ant aggressiveness against simulated herbivores, which increased in response to the highest EFN investment. Studies in facultative plant-ant mutualisms with induced EFN have also shown that the addition or removal of EFN clearly affects nectar-mediated mutualistic interactions [41], [44]. The application of artificial nectar increased the number of ants [45] and also reduced the herbivore damage [41]. However, EFN investment that has presumably evolved to attract defending ants may have the potential to attract antagonists as well. For example, in pollination mutualisms, illegitimate flower visitors can have significant costs to the plant by producing changes in the nectar quality and quantity that lead to changes in the foraging behaviour of pollinators [46], [47]. A higher investment of EFN leads usually to increased visits by other nectar consumers [41], which may cause ecological costs to plants by consuming this reward without exerting a beneficial effect. Does a higher EFN investment lead to an increase risk of exploitation by EFN thieves? And, what effect does this have on ant behaviour? We observed that ants of *P. ferrugineus* behaved more aggressively against EFN thieves when the concentration of nectar reward was higher, which significantly reduced the time spent on extrafloral nectaries by EFN thieves. Thus, a higher investment in EFN would not increase the risk of exploitation by non-ant consumers. This observation appears contradictory to the hypothesis that mutualisms have a higher chance of being exploited when the investment into the rewards increases [13], [48]. Why did more EFN, or EFN at higher concentrations, not increase its exploitation rate by nectar thieves? Several studies have suggested associations between ant aggressiveness and the availability of carbohydrates in the diet [49]–[51]. Carbohydrates would serve as a principal fuel for metabolically expensive behaviours, such as heightened aggression and activity in ants. This association probably explains our observation that a higher investment in EFN benefited the plant through an enhanced indirect defence against herbivores and an aggressive behaviour against EFN thieves.

For direct defences, there is strong evidence showing that differences in the quantity of secondary metabolites or physical traits influence the feeding behaviour of herbivores and damage levels caused by their activity [6], [52]–[54]. By contrast, for indirect defences, this quantitative relationship is much more difficult to prove, since the defence is provided by the attraction of the third trophic level, of which not all members are able to control herbivores [55], [56]. Our results provide quantitative information that EFN investment enhanced ant recruitment and was positively related to the ant-mediated protection against enemies. Moreover, *A. cornigera*, which invests more in the indirect defence than *A. hindsii*, gained stronger ant aggressiveness and defence against herbivores than did the latter species, and it did so particularly during the dry season, when the plants invest less in growth and relatively more in the - then more important - defence against herbivores. A higher investment in EFN, and consequently higher ant recruitment, can lead to significant fitness benefits in *Acacia* plants. Clement et al. [31] reported that the relative growth rates of *A. hindsii* increased with the relative degree of dominance by *P. ferrugineus*. Moreover, the chlorophyll content in *Acacia* plants is significantly affected by the removal of *P. ferrugineus* ants (González-Teuber, unpublished data).

EFN is an indirect defence, whose secretion might be controlled by plants in response to different consumers [57]. Here we showed that an increased plant investment into EFN enhanced the efficiency of ant-mediated defence and that this effect was not impaired by the presence of non-defending EFN thieves. Nevertheless, it still remains an open question whether a higher nectar production always leads to more service. Although there might be a point in nectar production that not necessarily results in a change in animal behaviour, a positive correlation of investment with benefit for the plant has been shown for EFN [41], [44], [45]. Thus, the indirect defence that is mediated by EFN is subject to a positive correlation between plant investment and better defence by ants and is therefore likely to be under a positive selection pressure by herbivores. Little is known on the selective pressures on nectar traits [58], although earlier studies have shown that the number and size of extrafloral nectaries can be under selective pressure exerted by herbivores and their enemies [59], [60]. Our observations now open in principle the possibility for a positive quantitative selection on nectar quantity as an important defensive trait. Further studies should consider different populations of EFN-secreting plant species growing under different levels of pressure by herbivores, in order to elucidate whether plants can adapt to these conditions with increased EFN secretion rates.
